# Fabrication of High Sensitivity Carbon Microcoil Pressure Sensors

**DOI:** 10.3390/s120810034

**Published:** 2012-07-25

**Authors:** Chih-Chung Su, Chen-Hung Li, Neng-Kai Chang, Feng Gao, Shuo-Hung Chang

**Affiliations:** 1 Department of Mechanical Engineering, National Taiwan University, Taipei 10617, Taiwan; E-Mails: r92522629@ntu.edu.tw (C.-C.S.); auko@hotmail.com (C.-H.L.); f90522632@ntu.edu.tw (N.-K.C.); 2 State Key Laboratory of Mechanical System & Vibration, School of Mechanical Engineering, Shanghai Jiao Tong University, 800 Dongchuan Road, Shanghai 200240, China; E-Mail: fengg@sjtu.edu.cn

**Keywords:** carbon microcoils, flexible sensor, chemical vapor deposition

## Abstract

This work demonstrates a highly sensitive pressure sensor that was fabricated using carbon microcoils (CMCs) and polydimethylsiloxane (PDMS). CMCs were grown by chemical vapor deposition using various ratios of Fe-Sn catalytic solution. The pressure sensor has a sandwiched structure, in which the as-grown CMCs were inserted between two PDMS layers. The pressure sensor exhibits piezo-resistivity changes in response to mechanical loading using a load cell system. The yields of the growth of CMCs at a catalyst proportion of Fe:Sn = 95:5 reach 95%. Experimental results show that the sensor achieves a high sensitivity of 0.93%/kPa from the CMC yield of 95%. The sensitivity of the pressure sensor increases with increasing yield of CMCs. The demonstrated pressure sensor shows the advantage of high sensitivity and is suitable for mass production.

## 1. Introduction

Pressure sensors, which are widely applied in automation equipment, robot arms, touch panels, and cell phones, have been developed on the basis of piezoresistive, piezoelectric, and capacitive principles [1–3]. Pressure sensors need high sensitivity and strong discriminatory abilities for wide applications. Various techniques using the Micro Electro Mechanical System (MEMS) processes and nanomaterials have been reported, such as the carbon nanotube pressure sensor [4,5] and carbon fiber pressure sensor [6]. However, these techniques require complex manufacturing processes and exhibit low piezoresistance sensitivity, less than 0.09%/kPa. Recently, carbon microcoils (CMCs) have been developed due to their unique 3-dimensional (3D) helical/spiral structure. The CMCs have been characterized for their mechanical properties [7–9], electrical properties, and magnetic properties [10–13]. Yang *et al.* found that carbon microcoils can be sensitive to very low applied loads on the order of milligrams, but the sensitivity of the sensor was not reported [14]. This work has developed a pressure sensor composed of a PDMS (polydimethylsiloxane)-CMCs-PDMS sandwich structure. The optimum ratio of Fe-Sn for the growth of CMCs has been determined. The sensitivity of the sensor was affected by the ratio of as-grown CMCs/carbon nanofibers (CNFs). This technique facilitates an increase in the sensitivity of a flexible pressure sensor using 3D helical CMCs and PDMS.

## 2. Experimental Section

**Figure 1.**
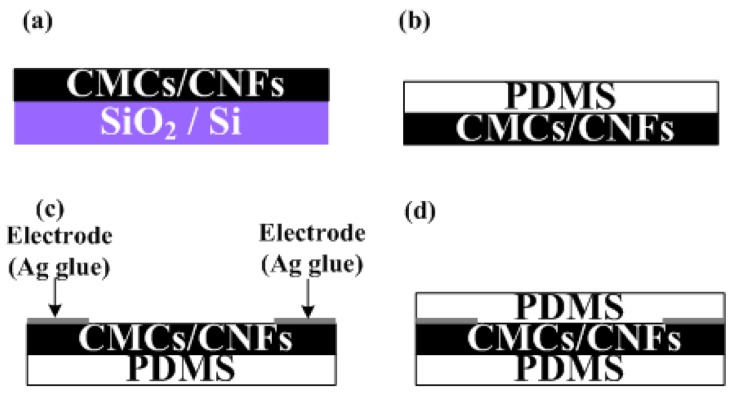
Fabrication process of CMC pressure sensor. (**a**) CMCs and CNFs were grown on SiO2 by CVD; (**b**) CMCs and CNFs were transferred on PDMS; (**c**) Ag glue was coated on both edges of the sample as electrode; (**d**) PDMS was coated on the Ag electrode to complete the CMC pressure sensor.

The fabrication process of the flexible pressure sensor is shown in Figure 1. The CMCs/CNFs were grown on silicon oxide film with dimensions of 10 × 10 mm^2^ by chemical vapor deposition (CVD) at 700 °C using ratios of Fe:Sn catalysts ranging from 80:20 to 97:3, as shown in Figure 1(a). A thickness of silicon oxide film of 500 nm was pre-deposited on 4 in silicon wafer (100) by a low-pressure chemical vapor deposition method. The powder catalyst for the CMCs grown herein was prepared by mixing Fe acetate and Sn acetate in Fe:Sn ratios = 80:20 to 97:3 in ethanol, wherein the molarity of Fe-Sn was 0.6 M. The substrate, a coating of Fe:Sn catalyst, was inserted into a quartz tube furnace at 450 °C in air for 30 min. Finally, the as-annealed sample was heated in a 1-inch quartz tube furnace at 700 °C under a 600 sccm flow of Ar gas. At 700 °C, C_2_H_2_ gas was supplied as the carbon source at a flow rate of 5 sccm for 15 min in order to grow CMCs. The PDMS was dropped on as-grown CMCs/CNFs in a cast to control the thickness of the PDMS layer. Simultaneously, the samples were placed in a low pressure chamber to eliminate the micro-bubbles inside the PDMS. After the PDMS was dried, both the PDMS and CMCs/CNFs were peeled off, as shown in Figure 1(b). Ag glue was coated on both edges of the samples as electrode (Figure 1(c)). When the Ag glue was dry, the top layer of PDMS was coated on CMCs/CNFs to complete the sensor, as shown in Figure 1(d). The dimensions of the pressure sensor in this work were 10 × 10 × 1 mm. A scanning electron microscope (SEM JEOL-6390) was used to examine the results of CMCs/CNFs. In addition, to evaluate the pressure sensing of the CMCs/CNFs pressure sensor, a load cell and multimeter were used to collect sensitivity measurements.

## 3. Results and Discussion

**Figure 2.**
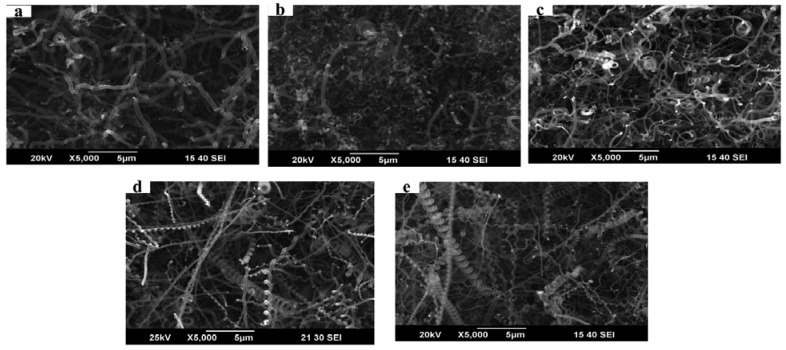
SEM images of the as-grown CMCs/CNFs from Fe-Sn powder catalyst at Fe-Sn mass ratios of (**a**) 80:20; (**b**) 85:15; (**c**) 90:10; (**d**) 95:5; and (**e**) 97:3.

Figure 2 presents SEM images of the morphology of the as-grown CMCs/CNFs at various Fe-Sn catalytic solutions. The mass ratio of Fe-Sn was controlled from 80:20 to 97:3 to determine the optimum proportion for the growth of CMCs. No CMCs were grown from the powder catalyst with a Fe-Sn ratio of 80:20. However, when the Fe-Sn ratio was controlled in the range of 95:5 to 97:3, CMCs were the main products. These results suggest that the optimal mass ratio of Fe-Sn was 95:5.

**Figure 3.**
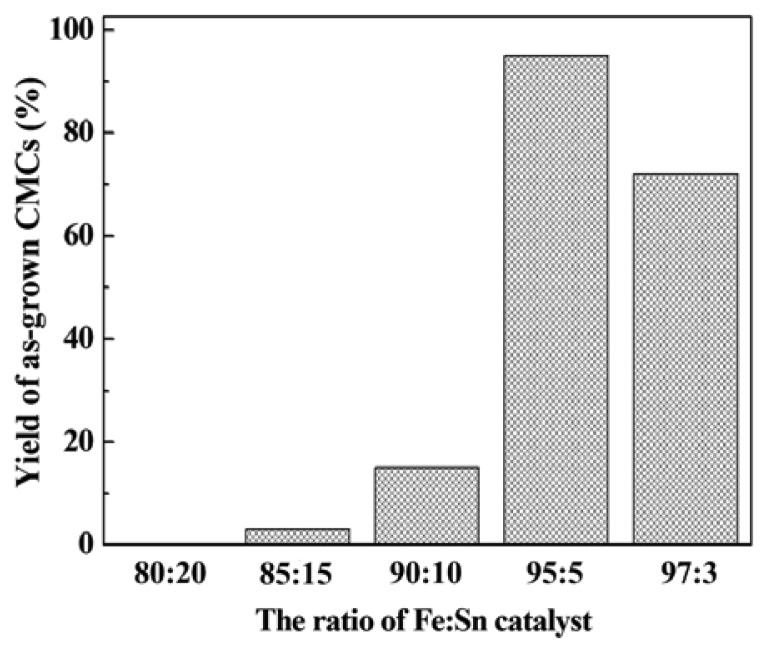
The yield ratio of as-grown CMCs *vs.* the different mass ratios of Fe-Sn catalytic solution.

Figure 3 shows the yield of CMCs *vs.* various ratios of Fe-Sn catalyst from 80:20 to 97:3. The yield of CMCs with the concentration of Fe-Sn of 95:5 achieved 95%. The yield was defined as the mass ratio of synthesis of CMCs to the amount of CMCs and CNFs, which was calculated from areas of 100 × 100 μm in 100 SEM images. These CMCs had a fiber diameter of 100 to 300 nm, a coil diameter of 100 to 1,000 nm, and a pitch of 200 to 1,200 nm. The appropriate composition ratio of Fe and Sn is critical for producing CMC structures. The amount of Sn should be reduced to maintain the correct ratio of Fe to Sn [15]. Energy dispersive X-ray analysis of the catalyst particles within the CNC tips showed the existence of Fe and Sn with a ratio of about 19:1, which is consistent with the results reported in [16]. Therefore, the concentration of Fe-Sn of 95:5 gives the greatest yield of CMCs. The growth mechanism of CMCs is believed to be due to the difference between the carbon diffusion and extrusion speeds in different parts of the catalyst comprising various metals [17].

**Figure 4.**
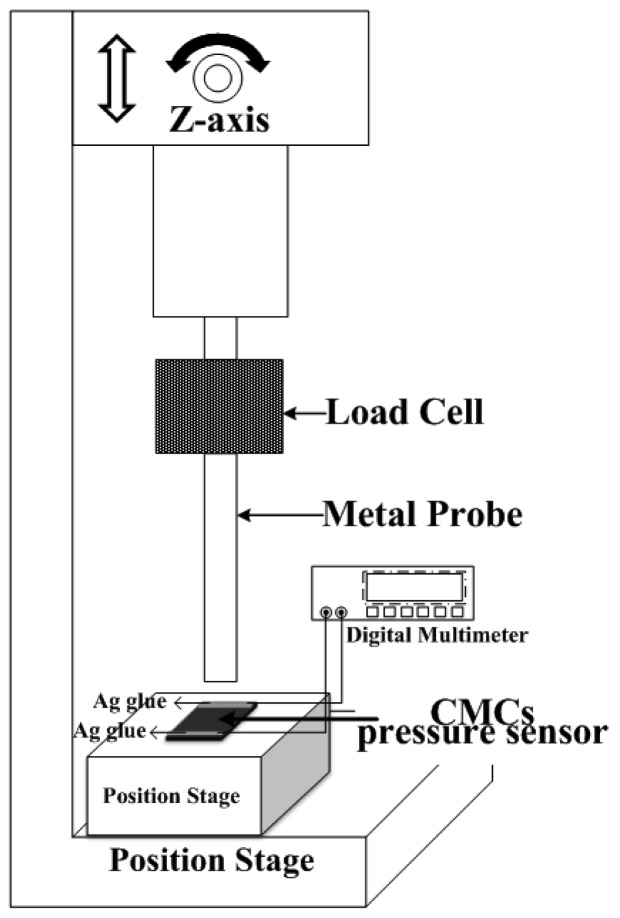
Experimental setup for measuring the pressure sensor of CMCs.

**Figure 5.**
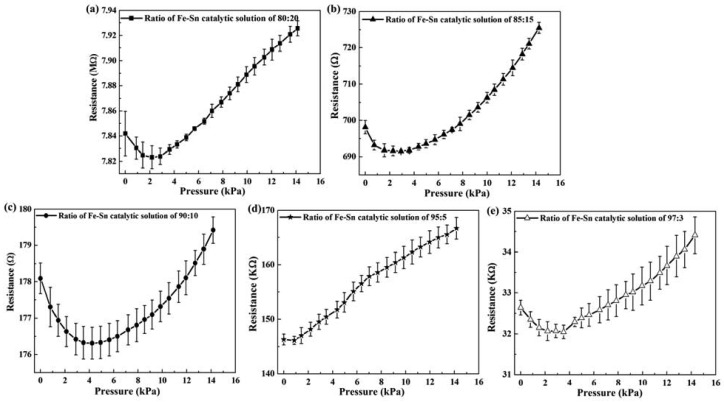
Relationship of resistivity *vs.* applied pressure for different mass ratios of Fe-Sn catalytic solution, (**a**) 80:20; (**b**) 85:15; (**c**) 90:10; (**d**) 95:5; and (**e**) 97:3.

Figure 4 is a schematic of the measurement setup for characterizing the pressure sensor of CMCs. The electrodes of Ag glue at both ends of the sample were connected to a multimeter for measurement of resistance. The corresponding resistivity of the CMCs could be evaluated by the measured resistance values under repeatable measurements (six times). The measured results of resistivity *vs.* applied pressure (0∼14 kPa) for the CMC pressure sensor with different yields of CMC growth are shown in Figure 5. Each point in the figure is the average value of one sample under 20 different applied pressures. The resistances increased with increases in the applied force from 3 to 14 kPa. The CMCs had higher resistance, suggesting that the helical CMCs affect the current transfer. Notably, the resistances decreased with increases in the applied force from 0 to 3 kPa. This result means that the CMCs/CNFs were not tightly connected, and some empty space exists between them. The CMCs/CNFs became dense and tight with increases in the applied force, resulting in increases in the conducting area and decreases in the electrical resistance. With continuous increases in the applied force from 3 to 14 kPa, the density of CMCs/CNFs mats did not increase, leading to an increase in the resistance. Interestingly, it can be seen that the resistance linearly increases with increasing applied force, as shown in Figure 5(d); *i.e.*, the 3D structure of CMCs can apparently cause an increase in the resistance with a larger applied force. In addition, the distribution of as-grown carbon materials affected the contact resistance of the loading pressure. The resistance of the catalyst ratio of Fe-Sn=80:20 is on the order of mega-ohms. The results showed that the non-uniform as-grown CNFs of the two dimensional materials lead to large contact resistance after transferring on PDMS.

The resistance decreased with increases in the as-grown CMCs, as shown in Figure 5. Therefore, the contact areas of the CNFs were smaller than those of the CMCs/CNFs. The variant resistances of Figure 5(b–d) should be from the non-uniform as-grown carbon materials. However, these results show the advantage of great resistance variation with the yield of 3D-structure CMCs in this work, resulting in high sensitivity.

**Figure 6.**
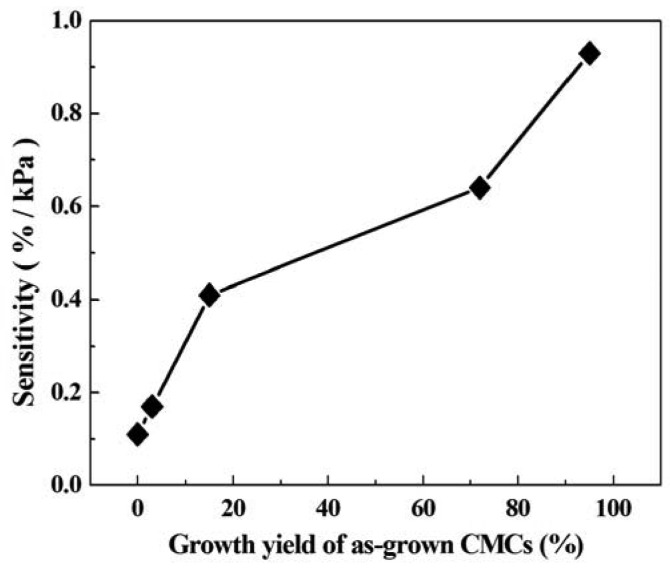
Sensitivity of CMC pressure sensor *vs.* the growth yield of as-grown CMCs.

The sensitivity of the CMC pressure sensor was defined thus: sensitivity of pressure sensor = ((ΔR/R_l_) × 100%)/ΔP, where ΔR = R_h_ − R_l_, R_h_ is the highest measured resistance, R_l_ is the lowest measured resistance, ΔP = P_h_ − P_l_, P_h_ is the applied pressure of the highest measured resistance, and P_l_ is the applied pressure of the lowest measured resistance. Figure 6 shows the sensitivity of the CMC pressure sensor with different ratios of Fe-Sn catalyst. This result indicates that the Fe-Sn catalyst of 95:5 had a maximum sensitivity of 0.93%/kPa. The sensitivity of the CMC pressure sensor increased with increases in the yield of CMCs. As compared to other pressure sensors composed of micro-materials, the CMC pressure sensor in this work has the greatest sensitivity (Table 1). The sensitivity in this work is almost 10.3 times that reported in [4] (metallic single-walled carbon nanotube), 25.6 times that in [5] (multi-walled carbon nanotubes), and 15.1 times that in [6] (carbon fiber). The 3D structure of CMCs allowed a large amount of contact area, resulting in the greatest variation in contact resistance.

## 4. Conclusions

This work demonstrates a highly sensitive pressure sensor with a sandwiched structure of PDMS/CMCs/PDMS. The growth of CMCs was controlled with different ratios of Fe-Sn catalyst using CVD from acetylene at 700 °C. A yield of CMCs of 95% was achieved with a ratio of Fe-Sn catalyst of 95:5. It is clearly shown that the ratio of CMCs/CNFs in the sensor dramatically affects the sensing characterization. The sensitivity of the pressure sensor increases with increased yield of CMCs. The pressure sensor in this work can achieve a sensitivity of 0.93%/kPa. This result reveals the remarkable potential to assemble CMCs on flexible chips.

## Figures and Tables

**Table 1.** Comparison of sensitivity of pressure sensors using nano-materials.

**Reference**	**Sensing material**	**Sensitivity (%/kPa)**
[4]	Single-walled carbon nanotube with Al_2_O_3_ thin film	0.09
[5]	Multi-walled carbon nanotube with PMMA	0.035
[6]	Carbon fiber with SOI wafer	0.0618
**This work**	**CMCs with PDMS**	**0.93**
